# Identification of the promising mango (*Mangifera indica* L.) genotypes based on morphological and pomological characters

**DOI:** 10.1002/fsn3.2961

**Published:** 2022-07-18

**Authors:** Ali Khadivi, Farhad Mirheidari, Abdolvahid Saeidifar, Younes Moradi

**Affiliations:** ^1^ Department of Horticultural Sciences, Faculty of Agriculture and Natural Resources Arak University Arak Iran; ^2^ Ministry of Agriculture Jihad, Sistan‐va‐Baluchestan Iran

**Keywords:** breeding, cultivation, fruit quality, germplasm, *Mangifera indica* L.

## Abstract

Mango (*Mangifera indica* L.) is one of the choicest fruit crops of the tropical and subtropical regions in the world. Morphological and pomological diversity of 18 mango (*M. indica*) genotypes (with 3–10 replications for each genotype, 81 trees in total) was evaluated from four areas of Sistan‐va‐Baluchestan province, Iran. There were significant differences among the genotypes investigated based on the traits recorded. Harvest date ranged from late May to early August. Fruit skin ground color was highly variable, including light green, green, light yellow, yellow, and orange. The values of fruit dimensions‐related characters were as follows: fruit length: 45.67–142.21 mm, fruit diameter: 37.51–94.13 mm, and fruit weight: 44.58–469.42 g. Peel and pulp percentages ranged from 65.24 to 92.45%. The quantity of fiber on stone was intermediate in most of the genotypes. Fruit weight showed positive standardized beta‐coefficient (*β*) values with stone weight (*β* = 0.66, *p* < .00) and pulp and skin content (*β* = 0.44, *p* < .00). Thus, these two key variables are the main traits accounting for fruit weight, and they should be considered together in breeding programs. Principal component analysis (PCA) showed 21 components explaining 85.44% of the total variance, and the first principal component (PC1) was positively correlated with fruit‐related traits. A dendrogram created using Euclidean distances and the Ward's method revealed two main clusters. High dissimilarity levels among the studied genotypes showed high variability in the germplasm. Based on the traits related to fruit quality, seven genotypes, including GulabKhas, Chaunsa, Ghalami, Soldan, Porteghali, KalmiBozorg, and Jangal, were superior and are recommended to use for cultivation in commercial orchards for area‐specific and in breeding programs.

## Graphical abstract

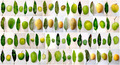

## 1 INTRODUCTION

Mango (*Mangifera indica* L.) is one of the choicest fruit crops of the tropical and subtropical regions in the world. Its popularity and importance can easily be realized by the fact that it is referred to as the “king of fruits” in the tropical world (Dinesh & Vasugi, 2002). The increasing demand for the mango fruit is due to the fruit's high vitamin, mineral, and fiber levels besides the value‐added products made from it. Consequently, the fruit brings economic benefits from both local sales and foreign earnings upon export (HCDA, 2010). Mango is also a particularly rich source of polyphenols, a diverse group of organic micronutrients found in plants which exert specific health benefits (Shahidi et al., 1992). Polyphenols identified in mango mesocarp include mangiferin, gallic acid, gallotannins, quercetin, isoquercetin, ellagic acid, and *β*‐glucogallin (Berardini et al., 2004, 2005), with gallic acid being the most represented phenol compound in this fraction. Furthermore, up to 25 diverse carotenoids have been identified in the mesocarp fraction, such as provitamin A, lutein, *α*‐carotene, and *β*‐carotene that account for the yellowish color of this part of the fruit (Masibo & He, 2008).

Mango has been reported to have extensive diversity due to alloploidy, outbreeding, repeated grafting, and phenotypic differences arising from varied agroclimatic conditions in different mango growing regions (Ravishankar & Lalitha, 2000). The important commercial mango varieties introduced in several countries remain to be fully characterized and adopted for cultivation in different regions. In addition, cross‐pollination in mango could have resulted in new varieties not yet documented (Griesbach, 2003). Subsequently, the characterization of mango varieties has experienced great confusion in nomenclature with many synonyms existing for the same varieties. Further, while geneticists and plant breeders are particularly interested in diversity at the molecular level, agronomists are more concerned with how visible morphological and agronomic variations can be used for sustainable farming (Hawkes, 1991). Also, farmers are faced with the challenge of identifying cultivars that are productive for their agroecological zones because they are unfamiliar with the characteristics of the many different cultivars of mango that are now grown and available in the country, resulting in lower productivity (Griesbach, 2003; Kehlenbeck et al., 2010; Wahdan & Abdelsalam, 2011).

Utilization of the conserved germplasm in the breeding programs requires precise information on the genetic relationships among the accessions. Information on the genetic distance among the germplasm accessions will also help avoid duplicates, thus clearing the nomenclature ambiguity, widening the genetic base of the core collections and ultimately helping in preservation of the valuable diversity (Rajan & Negi, 1999). Morphological characterization is a simple, formal, and standardized method of identifying and presenting genetic diversity. Assessment of morphological variation in fruit crops usually requires the availability of fruits. The fruiting season is unfortunately limited for most fruit crops. However, even in the off‐fruiting season, farmers, grafters, nursery managers, and breeders still require to discriminate varieties in such times as during selection and discrimination of rootstock or even during artificial pollination. This necessitates the identification of vegetative descriptors that can be used in the absence of fruits (Griesbach, 2003).

Little information is available on morphological and pomological traits for mango germplasm in Iran. Thus, the main objectives of the present research work were to characterize and evaluate morphological and fruits characteristics of the indigenous popular mango genotypes cultivated in Sistan‐va‐Baluchestan province, Iran. Characterization, evaluation, and documentation system for the studied germplasm will be invaluable for manipulating management of mango landraces, production, genetic conservation, and further breeding program for sustainable improvement of this crop.

## 2 MATERIALS AND METHODS

### 2.1 Plant material

Morphological and pomological diversity of 18 mango (*M. indica*) genotypes (with 3–10 replications for each genotype, 81 trees in total) was evaluated from four areas of Sistan‐va‐Baluchestan province, Iran, including Parood (located at 26°25'45“N latitude, 61°17'13”E longitude, and 595 m height above sea level), Markazi (located at 26°13′51“N latitude, 61°23'58”E longitude, and 389 m height above sea level), Pishin (located at 26°09′18“N latitude, 61°47'05”E longitude, and 243 m height above sea level), and Holonchegan (located at 26°17'19“N latitude, 60°43'40”E longitude, and 571 m height above sea level).

### 2.2 The characters evaluated

In total, 82 morphological and pomological variables were applied to investigate phenotypic variability among the genotypes (Table 1). Morphological and pomological evaluations were carried out using 50 replications of leaves and fruits per genotypes. The dimensions of leaf, fruit, stone, and seed were measured using a digital caliper. The weight of fruit, stone, and seed was measured using an electronic balance with 0.01 g precision. The remaining characters were qualitatively measured using rating and coding (Table 2) according to the mango guidelines descriptor (IPGRI, 2006).

**TABLE 1:** Statistical descriptive parameters for morphological traits used to study *M. indica* genotypes

No.	Traits	Unit	Min.	Max.	Mean	SD	CV (%)
1	Tree growth habit	Code	1	5	2.70	0.96	35.56
2	Tree vigor	Code	1	5	4.42	1.07	24.10
3	Tree height	Code	1	7	4.15	1.82	43.90
4	Crown diameter	Code	1	5	4.32	1.10	25.46
5	Crown shape	Code	1	7	5.42	2.03	37.45
6	Canopy density	Code	1	5	3.82	1.41	36.96
7	Shoot color	Code	1	7	4.55	1.96	43.10
8	Branching	Code	1	5	4.00	1.31	32.80
9	Branch density	Code	1	5	3.95	1.31	33.19
10	Branch flexibility	Code	1	5	2.90	1.23	42.34
11	Trunk type	Code	1	3	1.25	0.67	53.28
12	Trunk circumference	Code	1	5	3.35	1.55	46.33
13	Harvest date	Date	1	15	8.02	3.68	45.94
14	Yield	Code	1	5	3.15	1.49	47.14
15	Tree foliage density	Code	1	5	4.12	1.18	28.74
16	Leaf blade shape	Code	1	9	3.38	2.45	72.37
17	Leaf attitude in relation to branch	Code	1	5	3.85	1.34	34.86
18	Leaf texture	Code	1	3	1.68	0.95	56.67
19	Leaf apex shape	Code	1	5	4.38	1.08	24.75
20	Leaf base shape	Code	1	5	2.90	0.70	24.28
21	Leaf blade length	mm	95.83	327.42	189.17	51.05	26.99
22	Leaf blade width	mm	27.44	85.23	50.70	12.71	25.07
23	Form of leaf margin	Code	1	3	2.28	0.97	42.46
24	Color of fully developed leaf	Code	1	3	2.40	0.92	38.42
25	Leaf fragrance	Code	0	3	0.78	0.75	95.64
26	Petiole length	mm	8.87	53.62	27.23	10.40	38.20
27	Petiole thickness	mm	1.30	3.17	2.05	0.40	19.51
28	Thickness of pulvinus	mm	2.03	4.69	3.17	0.60	18.93
29	Petiole color	Code	1	5	2.68	1.40	52.35
30	Angle of secondary veins to the midrib	Code	1	5	2.97	1.25	42.19
31	Curvature of secondary veins	Code	0	1	0.76	0.43	56.32
32	Fruit length	mm	45.67	142.21	94.52	20.40	21.58
33	Fruit diameter	mm	37.51	94.13	63.93	11.61	18.16
34	Fruit weight	g	44.58	469.42	182.51	93.40	51.17
35	Fruit shape	Code	1	7	5.42	1.98	36.51
36	Fruit apex shape	Code	1	5	2.62	1.72	65.50
37	Fruit base shape	Code	1	5	2.42	1.11	45.91
38	Fruit attractiveness	Code	1	5	3.65	1.30	35.70
39	Fruit skin ground color	Code	1	9	4.15	2.75	66.31
40	Fruit skin blush	Code	1	5	2.18	1.30	59.63
41	Fruit skin thickness	mm	0.45	2.71	1.20	0.53	44.42
42	Fruit skin surface texture	Code	1	3	1.15	0.53	46.09
43	Density of lenticels on fruit skin	Code	1	5	3.18	1.47	46.07
44	Fruit pedicel width	mm	1.79	4.63	2.89	0.57	19.62
45	Fruit stalk insertion	Code	1	3	1.92	1.00	52.24
46	Depth of fruit stalk cavity	Code	0	3	0.94	1.19	127.02
47	Fruit stalk attachment	Code	1	5	3.00	1.27	42.43
48	Fruit neck prominence	Code	0	3	1.00	1.07	106.70
49	Slope of fruit ventral shoulder	Code	1	5	3.75	1.03	27.33
50	Fruit beak type	Code	1	7	2.97	1.92	64.71
51	Fruit sinus type	Code	0	3	0.92	0.91	99.02
52	Fruit skin waxiness	Code	0	1	0.60	0.49	82.17
53	Pulp color of ripe fruit	Code	1	7	5.35	1.82	34.06
54	Pulp texture of ripe fruit	Code	1	5	2.10	1.31	62.29
55	Adherence of fruit skin to pulp	Code	1	5	3.22	1.46	45.28
56	Quantity of latex oozing from peduncle	Code	1	5	2.60	1.29	49.58
57	Fruit pulp thickness	mm	5.95	29.92	17.10	4.38	25.63
58	Quantity of fiber in pulp	Code	1	5	2.45	1.56	63.59
59	Adherence of fiber to fruit skin	Code	1	5	2.20	1.55	70.27
60	Fiber length in the pulp	Code	1	5	2.15	1.45	67.44
61	Pulp and skin content	Ratio	65.24	92.45	80.15	5.48	6.84
62	Pulp juiciness	Code	1	5	3.95	1.27	32.20
63	Pulp aroma	Code	1	5	3.60	1.57	43.67
64	Presence of turpentine flavor	Code	0	5	1.61	1.63	100.99
65	Eating quality	Code	1	5	3.52	1.55	44.06
66	Stone length	mm	37.34	121.60	76.72	17.51	22.83
67	Stone width	mm	22.45	62.14	36.30	6.47	17.83
68	Stone thickness	mm	4.56	28.44	19.92	3.68	18.47
69	Stone weight	g	5.64	61.74	33.12	11.92	35.99
70	Veins on stone	Code	1	5	3.00	1.27	42.43
71	Pattern of stone venation	Code	1	3	1.33	0.74	55.79
72	Quantity of fiber on stone	Code	1	5	3.20	1.34	41.72
73	Length of stone fiber	Code	1	5	3.18	1.57	49.21
74	Adherence of fiber to stone	Code	1	5	4.05	1.27	31.41
75	Texture of stone fiber	Code	1	3	1.18	0.57	48.22
76	Space occupied by seed inside the stone	Code	1	7	5.30	1.46	27.58
77	Seed length	mm	18.98	92.82	62.50	14.52	23.22
78	Seed width	mm	7.65	49.98	28.30	6.04	21.35
79	Seed thickness	mm	1.78	24.24	15.87	3.40	21.44
80	Seed weight	g	1.65	38.96	17.41	6.80	39.05
81	Seed shape	Code	1	3	2.35	0.94	40.13
82	Type of embryonic	Code	1	3	1.08	0.38	35.37

**TABLE 2:** Frequency distribution for the measured qualitative morphological characters in the studied *M. indica* genotypes

	Frequency (no. of genotypes)								
Character	0	1	3	5	7	9	11	13	15
Tree growth habit	—	Drooping (16)	Spreading (60)	Erect (4)	—	—	—	—	—
Tree vigor	—	Low (3)	Intermediate (17)	High (60)	—	—	—	—	—
Tree height	—	Short (9)	Medium (30)	Tall (27)	Very tall (14)	—	—	—	—
Crown diameter	—	Low (3)	Intermediate (21)	High (56)	—	—	—	—	—
Crown shape	—	Oblong (9)	Broadly pyramidal (7)	Semicircular (22)	Spherical (42)	—	—	—	—
Canopy density	—	Sparse (10)	Intermediate (27)	Dense (43)	—	—	—	—	—
Shoot color	—	Light brown (9)	Brown (22)	Dark brown (27)	Gray‐Brown (22)	—	—	—	—
Branching	—	Low (7)	Intermediate (26)	High(47)	—	—	—	—	—
Branch density	—	Sparse (7)	Intermediate (28)	Dense (45)	—	—	—	—	—
Branch flexibility	—	Low (17)	Intermediate (50)	High (13)	—	—	—	—	—
Trunk type	—	Single‐trunk (70)	Multitrunk (10)	—	—	—	—	—	—
Trunk circumference	—	Low (18)	Intermediate (30)	High (32)	—	—	—	—	—
Harvest date	—	Late May (2)	Early June (13)	Mid‐June (15)	Late June (7)	Early July (8)	Mid‐July (28)	Late July (4)	Early August (3)
Yield	—	Low (19)	Intermediate (36)	High (25)	—	—	—	—	—
Tree foliage density	—	Sparse (4)	Intermediate (27)	Dense (49)	—	—	—	—	—
Leaf blade shape	—	Elliptic (30)	Oblong (27)	Ovate (2)	Lanceolate (20)	Oblanceolate (1)	—	—	—
Leaf attitude in relation to branch	—	Semi‐drooping (8)	Horizontal (30)	Semi‐erect (42)	—	—	—	—	—
Leaf texture	—	Coriaceous (53)	Chartaceous (27)	—	—	—	—	—	—
Leaf apex shape	—	Obtuse (3)	Acute (19)	Acuminate (58)	—	—	—	—	—
Leaf base shape	—	Obtuse (7)	Acute (70)	Round (3)	—	—	—	—	—
Form of leaf margin	—	Entire (29)	Wavy (51)	—	—	—	—	—	—
Color of fully developed leaf	—	Green (24)	Dark green (56)	—	—	—	—	—	—
Leaf fragrance	Absent (28)	Mild (47)	Strong (5)		—	—	—	—	—
Petiole color	—	Light green (27)	Green (39)	Dark green (14)	—	—	—	—	—
Angle of secondary veins to the midrib	—	Narrow (16)	Medium (49)	Wide (15)	—	—	—	—	—
Curvature of secondary veins	Absent (19)	Present (61)	—	—	—	—	—	—	—
Fruit shape	—	Oblong (8)	Elliptic (8)	Roundish (23)	Obovoid (41)	—	—	—	—
Fruit apex shape	—	Obtuse (38)	Acute (18)	Round (23)	—	—	—	—	—
Fruit base shape	—	Truncate (27)	Covex (49)	Angular (4)	—	—	—	—	—
Fruit attractiveness	—	Low (8)	Intermediate (38)	High (34)	—	—	—	—	—
Fruit skin ground color	—	Light green (25)	Green (19)	Light yellow (6)	Yellow (25)	Orange (5)	—	—	—
Fruit skin blush	—	Yellow (40)	Orange (33)	Red (7)	—	—	—	—	—
Fruit skin surface texture	—	Smooth (74)	Rough (6)	—	—	—	—	—	—
Density of lenticels on fruit skin	—	Low (18)	Intermediate (37)	High (25)	—	—	—	—	—
Fruit stalk insertion	—	Vertical (43)	Oblique (37)	—	—	—	—	—	—
Depth of fruit stalk cavity	Absent (41)	Shallow (21)	Medium (18)	—	—	—	—	—	—
Fruit stalk attachment	—	Weak (16)	Intermediate (48)	Strong (16)	—	—	—	—	—
Fruit neck prominence	Absent (30)	Slightly prominent (35)	Prominent (15)	—	—	—	—	—	—
Slope of fruit ventral shoulder	—	Slopping abruptly (1)	Ending in a long curve (48)	Rising and then rounded (31)	—	—	—	—	—
Fruit beak type	—	Perceptible (29)	Pointed (31)	Prominent (12)	Mammiform (8)	—	—	—	—
Fruit sinus type	Absent (26)	Shallow (44)	Deep (10)	—	—	—	—	—	—
Fruit skin waxiness	None‐waxy (32)	Waxy (48)	—	—	—	—	—	—	—
Pulp color of ripe fruit	—	Light yellow (3)	Yellow (18)	Yellow orange (21)	Orange (38)	—	—	—	—
Pulp texture of ripe fruit	—	Soft (43)	Intermediate (30)	Firm (7)	—	—	—	—	—
Adherence of fruit skin to pulp	—	Weak (17)	Intermediate (37)	Strong (26)	—	—	—	—	—
Quantity of latex oozing from peduncle	—	Low (26)	Medium (44)	High (10)	—	—	—	—	—
Quantity of fiber in pulp	—	Low (38)	Medium (26)	High (16)	—	—	—	—	—
Adherence of fiber to fruit skin	—	Low (46)	Medium (20)	High (14)	—	—	—	—	—
Fiber length in the pulp	—	Short (45)	Medium (24)	Long (11)	—	—	—	—	—
Pulp juiciness	—	Slightly juicy (6)	Juicy (30)	Very juicy (44)	—	—	—	—	—
Pulp aroma	—	Mild (16)	Intermediate (24)	Strong (40)	—	—	—	—	—
Presence of turpentine flavor	Absent (23)	Mild (30)	Intermediate (18)	Strong (9)	—	—	—	—	—
Eating quality	—	Low (16)	Medium (27)	High (37)	—	—	—	—	—
Veins on stone	—	Level with surface (16)	Depressed (48)	Elevated (16)	—	—	—	—	—
Pattern of stone venation	—	Parallel (67)	Forked (13)	—	—	—	—	—	—
Quantity of fiber on stone	—	Low (14)	Intermediate (44)	High (22)	—	—	—	—	—
Length of stone fiber	—	Short (21)	Medium (31)	Long (28)	—	—	—	—	—
Adherence of fiber to stone	—	Weak (6)	Intermediate (26)	Strong (48)	—	—	—	—	—
Texture of stone fiber	—	Soft (73)	Coarse (7)	—	—	—	—	—	—
Space occupied by seed inside the stone	—	≤ 25% (3)	26–50% (7)	51–75% (45)	76–100% (25)	—	—	—	—
Seed shape	—	Oblong (26)	Reniform (54)	—	—	—	—	—	—
Type of embryonic	—	Monoembryonic (77)	Polyembryonic (3)	—	—	—	—	—	—

### 2.3 Statistical analysis

Analysis of variance (ANOVA) was performed to evaluate the variation among genotypes based on the traits measured using SAS software (SAS Institute, Cary, NC, USA, 1990). Simple correlations between traits were determined using Pearson correlation coefficients (Norusis, 1998). Principal component analysis (PCA) was used to investigate the relationship between genotypes and determine the main traits effective in genotype segregation using SPSS software. Hierarchical cluster analysis (HCA) was performed using Ward's method and Euclidean coefficient using PAST software (Hammer et al., 2001). The first and second principal components (PC1/PC2) were used to create a scatter plot with PAST software. Besides, independent traits affecting the fruit weight as a dependent trait were determined through multiple regression analysis (MRA) using the “linear stepwise” method with SPSS software.

## 3 RESULTS AND DISCUSSION

There were significant differences among the genotypes investigated based on the traits recorded. Also, the CV was more than 20.00% in 75 out of 82 characters measured, indicating high diversity among the genotypes. Depth of fruit stalk cavity showed the highest CV (127.02%), followed by fruit neck prominence (106.70%), presence of turpentine flavor (100.99%), fruit sinus type (99.02%), leaf fragrance (95.64%), fruit skin waxiness (82.17%), leaf blade shape (72.37%), and adherence of fiber to fruit skin (70.27%). In contrast, the lowest CV belonged to pulp and skin content (6.84%) (Table 1).

Tree growth habit was predominantly spreading (60 genotypes). Tree height was short (≤6.00 mm) in nine, medium (6.10–9.00 mm) in 30, tall (9.10–12.00 mm) in 27, and very tall (>12.00 m) in 14 genotypes. The value of tree vigor, crown diameter, canopy density, branching, and ranch density was high (Table 2). Varietal differences in plant growth were noted by Joshi et al. (2013). This variation among different genotypes might be due to the variation in genetic constitution and interaction of various genotypes with agroclimatic conditions. Similar results have been reported in mango varieties (Barua et al., 2013; Bhamini et al., 2018; Christopher et al., 2017; Fivaz, 2008; Majumder et al., 2011; Mitra et al., 2013). The vegetative characters related to tree emerge as important plant functional traits, even for fruit trees, because of their role for stability, defense, architecture, hydraulics, carbon gain, and growth potential, hence justifying this experiment (Bhamini et al., 2018).

Leaf blade shape was elliptic (30 genotypes), oblong (27), ovate (2), lanceolate (20), and oblanceolate (1). Leaf texture was coriaceous (53 genotypes) and chartaceous (27). Leaf apex shape was predominantly acuminate (58), while leaf base shape was predominantly acute (70) (Table 2). The value of leaf dimensions‐related characters was as follows: leaf blade length: 95.83–327.42 mm, leaf blade width: 27.44–85.23 mm, petiole length: 8.87–53.62 mm, petiole thickness: 1.30–3.17 mm, and thickness of pulvinus: 2.03–4.69 mm (Table 1). The variation in foliage density of mango genotypes might be due to variation in genetic constitution among different genotypes which confirms the earlier findings in mango (Bhamini et al., 2018; Dhillon et al., 2004). Characterization and identification of mango cultivars using leaf length, width, shape, apex, and base have been reported by numerous scientists (Barholia & Yadav, 2014; Bhamini et al., 2018; Khan et al., 2015; Mussane, 2010; Rajwana et al., 2011; Sennhenn et al., 2014).

Harvest date ranged from late May to early August. Yield was low (19 genotypes), intermediate (36), and high (25). Fruit shape was oblong (8 genotypes), elliptic (8), roundish (23), and obovoid (41). Fruit apex shape was obtuse in the majority of genotypes (38), while fruit base shape was covex in most of the genotypes (49). Fruit skin ground color was highly variable, included light green (25 genotypes), green (19), light yellow (6), yellow (25), and orange (5). Fruit color at maturity depends upon genotypes (Barholia & Yadav, 2014; Bhamini et al., 2018; Sennhenn et al., 2014). High variations have been found in fruit color as it varies from orange, yellow, green, or red at ripening stage (Barholia & Yadav, 2014; Rajwana et al., 2011; Sennhenn et al., 2014). Fruits generally have a dark green background that becomes light green to yellow in color as they ripe (Khan et al., 2015). Red blush may develop in some fruits at fruit set which may persist until the fruit ripe. The red blush in mango skin is also genotype dependent due to a pigment known as anthocyanin (Bally, 2006; Khan et al., 2015; Lizada, 1991).

Fruit skin surface texture was predominantly smooth (74 genotypes). Pulp color of ripe fruits showed strong variability and included light yellow (3), yellow (18), yellow orange (21), and orange (38). Pulp texture of ripe fruits was soft (43 genotypes), intermediate (30), and firm (7).

Quantity of fiber in pulp was low in 38, medium in 26, and high in 16 genotypes. Fiber length in the pulp was short (<1.00 cm) in 45, medium (1.00–1.50 cm) in 24, and long (>1.50 cm) in 11 genotypes. Pulp was very juicy in the majority of genotypes (44). Pulp aroma was mild (16 genotypes), intermediate (24), and strong (40), while eating quality was low (16 genotypes), medium (27), and high (37). Peel and pulp percentage ranged from 65.24 to 92.45%. The values of fruit dimensions‐related characters were as follows: fruit length: 45.67–142.21 mm, fruit diameter: 37.51–94.13 mm, fruit weight: 44.58–469.42 g, fruit skin thickness: 0.45–2.71 mm, fruit pedicel width: 1.79–4.63 mm, and fruit pulp thickness: 5.95–29.92 mm (Table 1). Similar results were reported by different workers (Bhamini et al., 2018; Chatterjee et al., 2005; Gupta & Brahmachari, 2004; Mannan et al., 2003). These variations might be due to the location enjoying different types of environmental conditions, year of production, and out crossing among different varieties (Bhamini et al., 2018; Mannan et al., 2003). The topic of fruit growth and development may be influenced by genes, proteins as well as agronomic practices, climate, and other mechanical processes that specify or affect the fruit formation and development. Plants compromised in photosynthesis, phloem transport, floral initiation and development, or male or female fertility either cannot produce fruit or are abnormal in their fruit production, that is, parthenocarpic fruit, reduced fruit size, or reduced fruit load (Tanksley, 2004). Khan et al. (2015) also reported that even if a variety of mango being grown in the same region, its quality will be affected by different environmental conditions.

Quantity of fiber on stone was low (14 genotypes), intermediate (44), and high (22), while length of stone fiber was short (21 genotypes), medium (31), and long (28). The value of stone dimensions‐related characters was as follows: stone length: 37.34–121.60 mm, stone width: 22.45–62.14 mm, stone thickness: 4.56–28.44 mm, and stone weight: 5.64–61.74 g.

**FIGURE 1:**
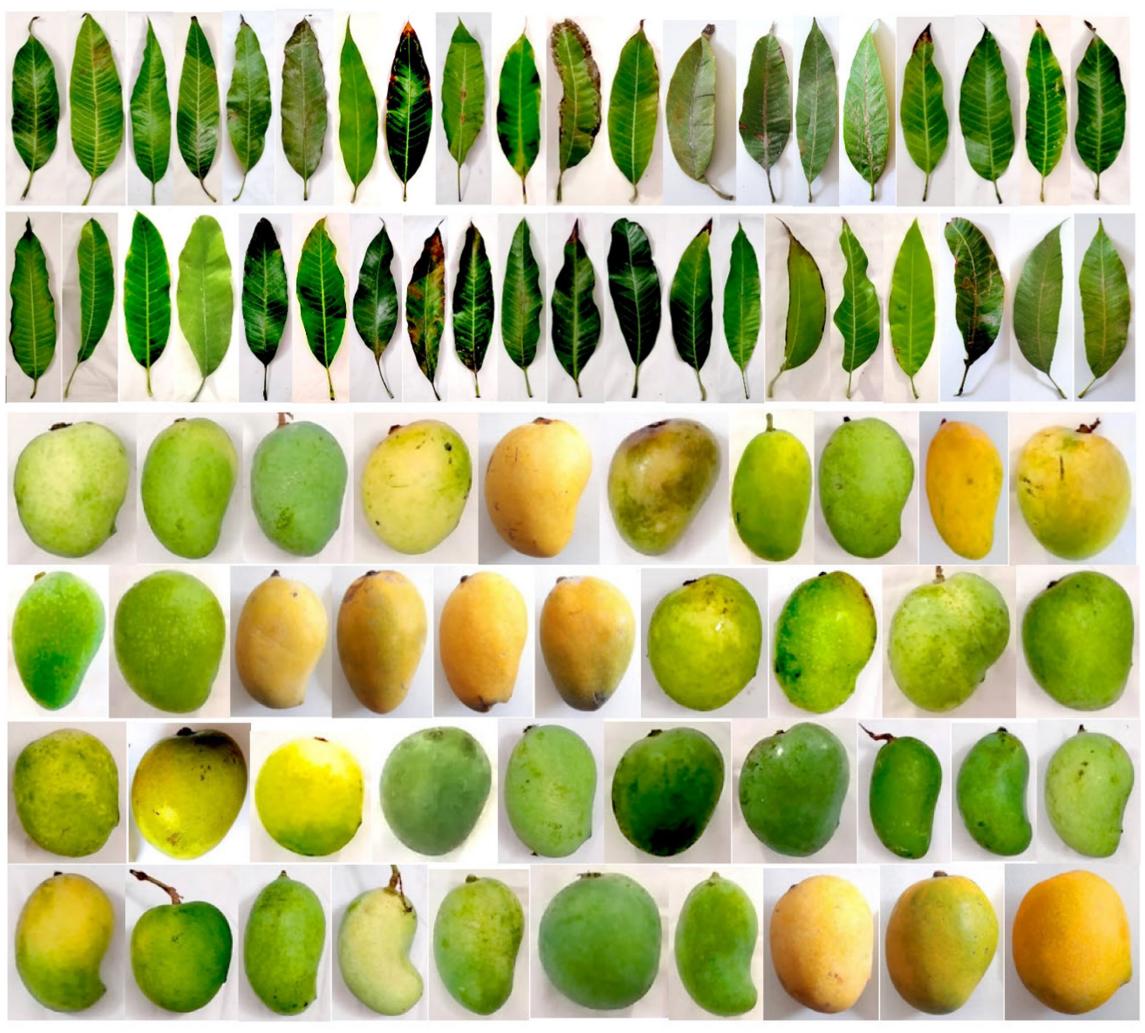
The pictures of leaves and fruits of *M. indica* genotypes studied

The values of seed dimensions‐related characters was as follows: seed length: 18.98–92.82 mm, seed width: 7.65–49.98 mm, seed thickness: 1.78–24.24 mm, and seed weight: 1.65–38.96 g. Seed shape was oblong (26 genotypes) and reniform (54). The majority of genotypes were monoembryonic (77) (Table 2). Seed may be monoembryonic or polyembryonic. Phenomenon of polyembryony has been found in 59 families, 158 genera and about 239 species (Khan et al., 2015; Tisserat et al., 1979). Nucellor embryony is also present in mango discovered in 19 mango cultivars (Khan et al., 2015; Sachar & Chopra, 1957). Adventive embryony (adventitious embryogenesis) established by the influence of single or more genes has been observed in mango. Eastern Indian cultivars consist of monoembryonic variations due to presence of overriding genes, while Cochin, China, Philippines, and Sunda Islands are polyembryonic due to the impact of recessive genes (Khan et al., 2015; Prasad & Prasad, 1972). The presence of polyembryony or monoembryony traits is most critical in germplasm characterization. So, mango cultivars have been classified into two major categories depending upon the type of embryo, that is, monoembryony and polyembryony (Iyer & Degani, 1997; Khan et al., 2015). Monoembryonic mango seed consists of only one embryo, usually having lobed shape and unequal in size; whereas, polyembryonic seed contains more than one embryo, among them one is zygotic and others arise from nucellus. The pictures of different organs of *M. indica* genotypes studied are shown in Figure 1.

After calculating the simple correlation coefficients, fruit weight was considered as a dependent variable, and then the direct and indirect effects of each independent variable on this key trait were calculated using MRA. The MRA showed that fruit weight was found to be associated with 18 characters (Table 3). Fruit weight showed positive standardized beta‐coefficient (*β*) values with stone weight (*β* = 0.66, *P* < 0.00) and pulp and skin content (*β* = 0.44, *P* < 0.00). Thus, these two key variables are the main traits accounting for fruit weight, and they should be considered together in breeding programs. An understanding association between these traits can help breeders for selection and crosses (Khadivi‐Khub & Ebrahimi, 2015).

**TABLE 3:** The traits associated with fruit weight in the *M. indica* genotypes as revealed using MRA and coefficients

Dependent character	Independent character	*r*	*r* ^2^	*β*	t value	*P* value
Fruit weight	Stone weight	.857 a	.734	.66	20.52	.00
	Pulp and skin content	.966 b	.933	.44	24.75	.00
	Fruit pedicel width	.972 c	.945	−.13	−7.84	.00
	Fruit skin waxiness	.976 d	.953	−.05	−2.64	.01
	Angle of secondary veins to the midrib	.979 e	.958	−.13	−7.51	.00
	Fruit skin blush	.981 f	.962	.12	6.62	.00
	Fruit sinus type	.982 g	.965	−.06	−3.08	.00
	Petiole length	.984 h	.968	.08	4.30	.00
	Trunk type	.986 i	.972	−0.12	−6.75	.00
	Form of leaf margin	.987 j	.974	.15	8.18	.00
	Stone thickness	.988 k	.977	.11	4.29	.00
	Branch density	.990 L	.98	.07	4.50	.00
	Petiole color	.991 m	.982	.06	3.48	.00
	Harvest date	.992 n	.983	.09	4.28	.00
	Adherence of fruit skin to pulp	.993 o	.985	−.04	−2.30	.03
	Yield	.994 p	.985	−.07	−3.58	.00
	Quantity of fiber on stone	.995 q	.986	.06	3.34	.00
	Stone length	.996 r	.987	.07	2.67	.01

The most important variables influencing to distinguish the variations among the genotypes were determined using the PCA. Eigenvalues greater than 1.00 were highlighted as criteria to extract the main components, to determine the PCs that showed the greatest value of diversity. The loaded values ≥0.53 were considered significant for each factor, which showed 21 components with explaining 85.44% of the total variance (not shown). The PC1 was positively correlated with fruit length, fruit diameter, fruit weight, fruit attractiveness, fruit pulp thickness, stone length, stone width, stone thickness, stone weight, seed length, seed width, seed thickness, and seed weight, accounting for 12.45% of total variance. Thus, PC1 could be called as fruit‐related traits. Five traits, including leaf blade length, leaf blade width, petiole length, petiole thickness, and thickness of pulvinus were significantly and positively correlated with PC2, accounting for 6.01% of total variance. Thus, PC2 could be called as vegetative‐related traits. The PC3 was associated with quantity of fiber in pulp, adherence of fiber to fruit skin, fiber length in the pulp, and pulp juiciness, accounting for 5.35% of total variance.

**FIGURE 2:**
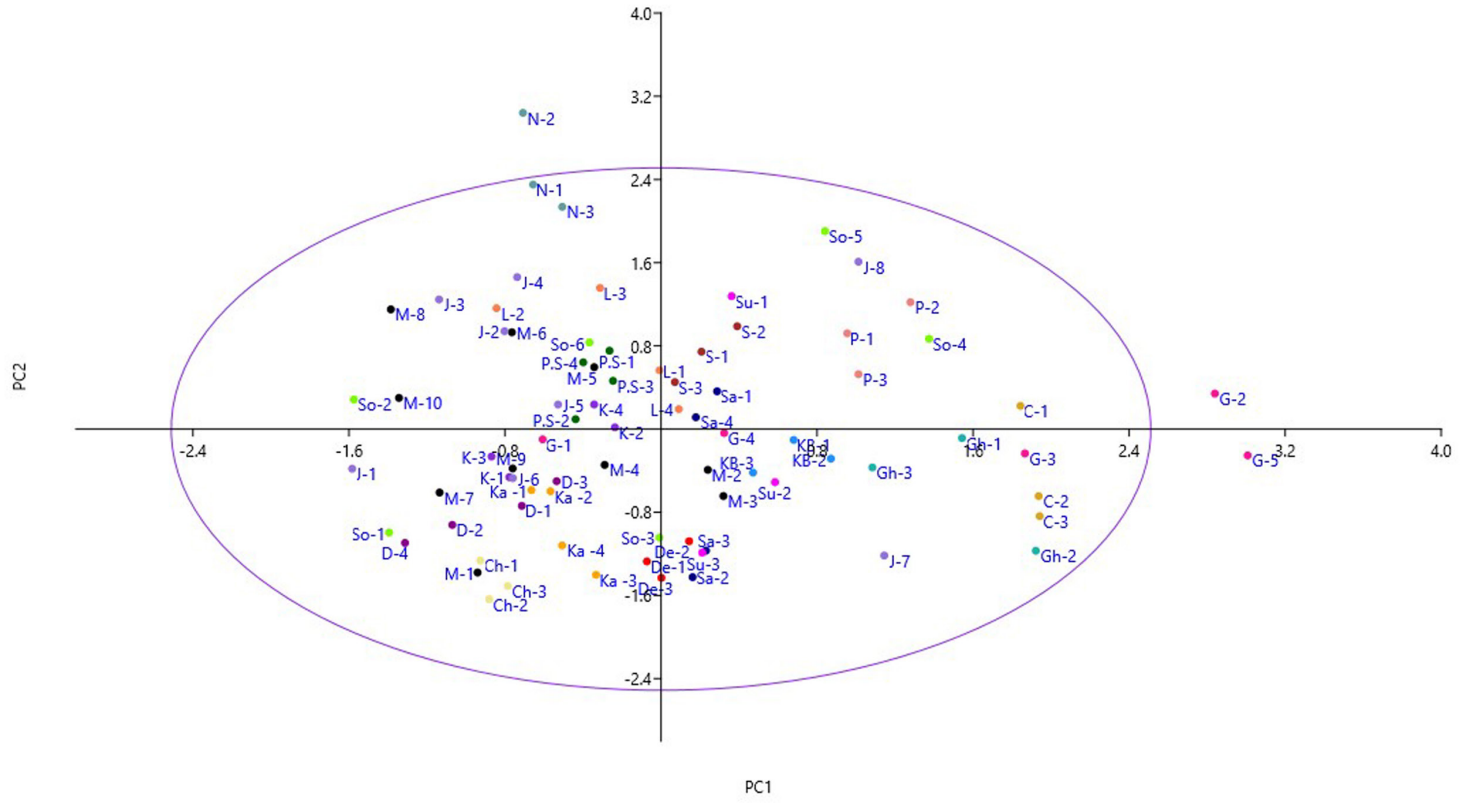
Scatter plot for the studied *M. indica* genotypes based on PC1/PC2. The symbols represent the genotypes in the plot, including Korch (K), Sandti (S), Nareh (N), Langra (L), Saroli (Sa), Padan‐Sarori (P.S), Dasheri (D), GulabKhas (G), Sundri (Su), Desi (De), Kalmi (Ka), KalmiBozorg (KB), Chadgali (Ch), Chaunsa (C), Porteghali (P), Ghalami (Gh), Maleki (M), Jangal (J), and Soldan (So)

The projection of the studied genotypes based on the PC1/PC2 plot reflected the relationship among them in terms of phenotypic resemblance (Figure 2). By starting from negative toward positive values of PC1, the genotypes showed gradual increases in fruit length, fruit diameter, fruit weight, fruit attractiveness, fruit pulp thickness, stone length, stone width, stone thickness, stone weight, seed length, seed width, seed thickness, and seed weight. Furthermore, starting from negative to positive values of PC2, the genotypes indicated gradual increases in leaf blade length, leaf blade width, petiole length, petiole thickness, and thickness of pulvinus.

**FIGURE 3:**
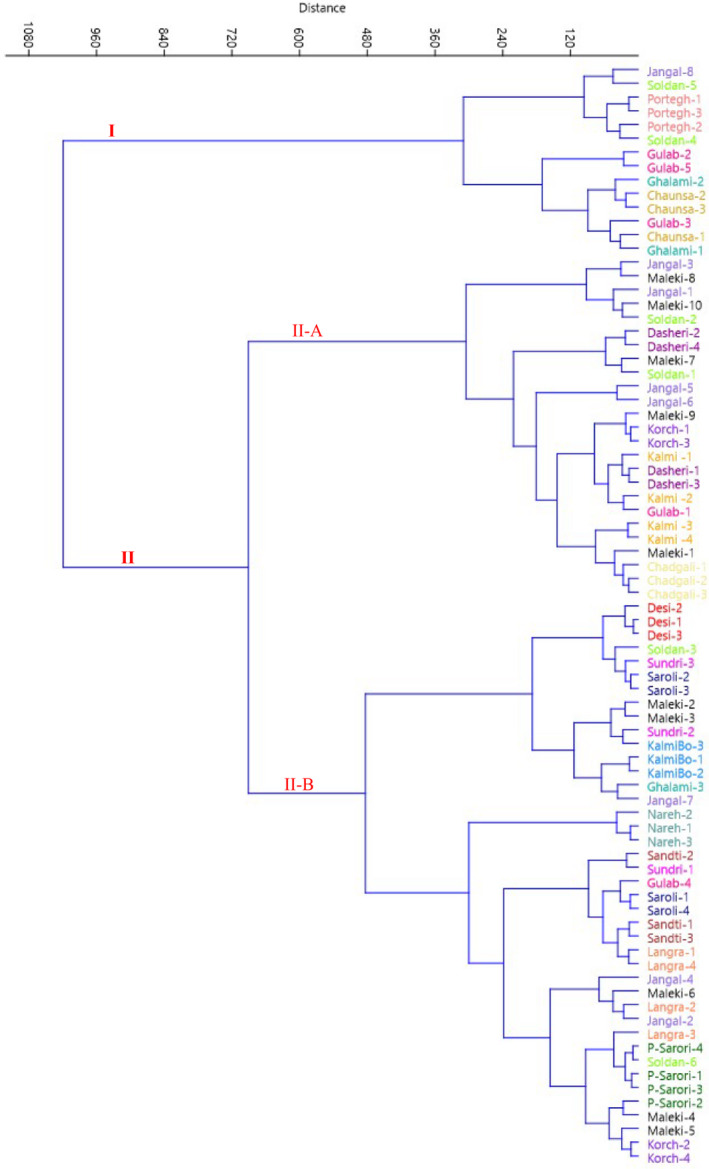
Ward cluster analysis of the studied *M. indica* genotypes based on morphological traits using Euclidean distances

A dendrogram created using Euclidean distances and the Ward's method revealed two main clusters (Figure 3). The first cluster (I) contained 14 genotypes. The second cluster (II) contained the rest genotypes, forming two subclusters. The subcluster II‐A contained 25 genotypes, while the remaining 41 genotypes formed subcluster II‐B. The obtained data revealed the morphological diversity within the studied genotypes.

High dissimilarity levels among the studied genotypes showed high variability in the germplasm. Morphological traits have been used traditionally to obtain information on variation within species. Continuous variation in fruit traits has important implications for domestication, suggesting opportunities for cultivar development through identification of elite individuals (Leakey & Page, 2006).

## 4 CONCLUSION

The information on genetic diversity and genetic relationship of the mango genotypes is very important to be documented for proper identification of superior genotypes. This knowledge can be used as a tool for plant breeders to improve the strategies in breeding programs as well as an initial effort to establish the conservation programs. Thus, an adequate study of local collections must be conducted before the accessions are lost. To prevent the loss, the farmers should be educated to maintain the valuable materials they possessed in their home garden or orchards. Therefore, consideration should be made to keep these accessions available in the germplasm collections for the future. Based on the traits related to fruit quality, seven genotypes, including GulabKhas, Chaunsa, Ghalami, Soldan, Porteghali, KalmiBozorg, and Jangal, were superior and are recommended to use for cultivation in commercial orchards and in breeding programs.

## FUNDING INFORMATION

None.

## CONFLICT OF INTEREST

The authors declare no conflict of interest.

## ETHICS STATEMENT

Research involving Human Participants and/or Animals

None.

## INFORMED CONSENT

None.

## Data Availability

The data that support the findings of this study are available from the corresponding author upon reasonable request.
